# A path towards SARS-CoV-2 attenuation: metabolic pressure on CTP synthesis rules the virus evolution

**DOI:** 10.1093/gbe/evaa229

**Published:** 2020-10-30

**Authors:** Zhihua Ou, Christos Ouzounis, Daxi Wang, Wanying Sun, Junhua Li, Weijun Chen, Philippe Marlière, Antoine Danchin

**Affiliations:** e1 BGI-Shenzhen, Shenzhen, China; e2 Shenzhen Key Laboratory of Unknown Pathogen Identification, BGI-Shenzhen, Shenzhen, China; e3 Biological Computation and Process Laboratory, Centre for Research and Technology Hellas, Chemical Process and Energy Resources Institute, Thessalonica, Greece; e4 BGI Education Center, University of Chinese Academy of Sciences, Shenzhen, China; e5 BGI PathoGenesis Pharmaceutical Technology, BGI-Shenzhen, Shenzhen, China; e6 TESSSI, The European Syndicate of Synthetic Scientists and Industrialists, 81 rue Réaumur, Paris, France; e7 Kodikos Labs, Institut Cochin, 24, rue du Faubourg Saint-Jacques Paris, France; e8 School of Biomedical Sciences, Li KaShing Faculty of Medicine, Hong Kong University, 21 Sassoon Road, Pokfulam, Hong Kong

**Keywords:** ABCE1, cytoophidia, Maxwell’s demon, Nsp1, phosphoribosyltransferase, queuine

## Abstract

In the context of the COVID-19 pandemic, we describe here the singular metabolic background that constrains enveloped RNA viruses to evolve towards likely attenuation in the long term, possibly after a step of increased pathogenicity. Cytidine triphosphate (CTP) is at the crossroad of the processes allowing SARS-CoV-2 to multiply, because CTP is in demand for four essential metabolic steps. It is a building block of the virus genome, it is required for synthesis of the cytosine-based liponucleotide precursors of the viral envelope, it is a critical building block of the host transfer RNAs synthesis and it is required for synthesis of dolichol-phosphate, a precursor of viral protein glycosylation. The CCA 3’-end of all the transfer RNAs required to translate the RNA genome and further transcripts into the proteins used to build active virus copies is not coded in the human genome. It must be synthesized de novo from CTP and ATP. Furthermore, intermediary metabolism is built on compulsory steps of synthesis and salvage of cytosine-based metabolites via uridine triphosphate (UTP) that keep limiting CTP availability. As a consequence, accidental replication errors tend to replace cytosine by uracil in the genome, unless recombination events allow the sequence to return to its ancestral sequences. We document some of the consequences of this situation in the function of viral proteins. This unique metabolic setup allowed us to highlight and provide a *raison d’être* to viperin, an enzyme of innate antiviral immunity, which synthesizes 3ʹ-deoxy-3′,4ʹ-didehydro-CTP (ddhCTP) as an extremely efficient antiviral nucleotide.

As COVID-19 expands its course, the genome of SARS-CoV-2 evolves. It is deficient in one of the four genomic bases, cytosine. Here we establish that when it multiplies, the virus taps into metabolic resources shaped by the availability of cytosine triphosphate (CTP), set up to be limiting to coordinate the cell's metabolism. This nucleotide is uniquely required not only for genome synthesis but also for synthesis of the virus envelope, translation (*via* addition of a CCA end to transfer RNA) and glycosylation of its proteins (*via* a terpene anchor binding to the endoplasmic reticulum). Innate antiviral immunity has evolved to generate a toxic analog of CTP, paving the way for the design of novel synthetic inhibitors of the virus development.

## INTRODUCTION

The COVID-19 pandemic motivated a deluge of literature investigating how the SARS-CoV-2 coronavirus develops and evolves. Molecular analyses of the virus' genome and of its proteins keep accumulating at a fast pace (https://viralzone.expasy.org/8996). Surprisingly, the way the virus taps into its cell host's metabolism to build up its genome, proteins and envelope is seldom explored. We investigated here how unique metabolic features impact on the virus' functions, aiming at understanding and possibly revealing conditions for alleviation of its virulence. Coronavirus genomes mimic the structure of cellular mRNAs, beginning with a conventional 5'-end methylated cap (Jin et al. 2013) and ending up with a 3'-polyadenylated tail. While remarkably apt to create a stealthy mRNA mimic, the SARS-CoV-2 genome is so similar to that of the host's mRNAs that standard interference with the virus expression machinery will often also interfere with that of non-infected cells and be toxic to the host. The virus is also an enveloped virus. Both of these attributes imply drawing resources from the cell's nucleotide and lipid metabolism.

A noteworthy feature shared by the viral envelope construction and genome synthesis is that both rely on cytosine triphosphate (CTP) availability. This prompted us to analyse the consequences of the nucleotide requirement for these processes, as compared to the host cell's metabolism that ends up as cellular mRNAs and membranes. We previously pointed out how a series of events which begins with copying the virus positive-sense RNA into a general template minus-sense RNA that serves to generate new viral genomes and several individual transcripts of that template (Sawicki et al. 2007; Chen et al. 2020) is tightly linked to the metabolism of cytosine-containing nucleotides (Danchin & Marlière 2020). We further develop here the singular role of CTP, in particular in its mandatory requirement for tRNA maturation into a functional entity, as this impacts availability of a functional translation machinery, exploring the phylogenetic consequences of this metabolic set-up. It had been noticed that the virus exploits a critical set of pyrimidine-related metabolic pathways to access the pool of ribonucleoside triphosphates needed for the RNA-dependent replication and transcription of the replicated RNA minus strand (Lucas-Hourani et al. 2013). However, the specific role of CTP was overlooked. In fact, CTP is used in four independent processes, all of them essential for the construction of active enveloped RNA viruses. CTP is required not only for (1) construction of the viral genome, but also for (2) the construction of a subset of lipid metabolism (Danchin & Marlière 2020), and, as developed here for the first time, (3) for synthesis of active tRNA molecules, and (4) for protein glycosylation via formation of dolichol-phosphate. This makes the viral sequence highly sensitive to metabolic details of the cell's CTP pool synthesis and maintenance, likely to be reflected in the virus evolution as it mutates with a slow general decrease in cytosine nucleotides, attributed at this time to causes that widely differ from what we present here—see e.g. (Xia 2020; Di Giorgio et al. 2020).

By contrast, functional analysis helped us to reveal unexpected key functions of the virus, marked by a divergent trend in the local content of cytosine nucleotides. For example, if the presence of a subset of amino acids—e.g. proline residues—in viral proteins was essential for key functions required for long term survival, then a local increase in cytosine residues would expand the evolutionary landscape of the virus. This type of local bias has indeed been highlighted in a previously discovered feature of coronavirus adaptation to the human host: in SARS-CoV-1 a GC-rich critical sequence of the virus spike protein—a leucine to alanine mutation derived from a GC local enrichment, with a UUA codon changed into GCA—

displayed positive selection in the course of evolution of the SARS disease in 2003 (Song et al. 2005). Here we depict first, with emphasis on SARS-CoV-2, the details of cytosine-based metabolism that must be retained as a unique coordinator of the global cell metabolism. We then explore the likely consequences of this dependency on the evolution both of cytosine-related innate immunity processes and of the viral genome sequence. Subsequently, we delineate critical details of the impact on the virus biological functions on the nucleotide composition of its genes and consequences for its short term and long term evolution.

**RESULTS**

**In-depth analysis of pyrimidine metabolism highlights the unique position of CTP in metabolism**

To understand how viruses recruit the functions of their host cells to their benefit, we must understand what would be the point of view of a virus if it were to sustain propagation over many generations. Essentially lacking biosynthetic potential, a virus must tap into the host's metabolic resources. This introduces a considerable limitation in the metabolic options offered to viral multiplication. For this reason many viruses ended up coding for functions that are missing or deficient in their hosts (Moreno-Altamirano et al. 2019). Some even help their hosts to upgrade their built-in ability to make the most of their environment, thus ensuring a wealthy propagation of the viral progeny. Auxiliary metabolic genes are commonplace in bacteriophages (Thompson et al. 2011), but also in a variety of eukaryotic viruses, such as herpes viruses (Hew et al. 2015). Selection pressure *via* efficacy of transmission multiplied by number of replicates per cell, coupled to selection stemming from intracellular availability of essential precursors (nucleotides, amino acids, lipids and carbohydrates) creates a variety of bottlenecks that shape the virus evolutionary landscape (Kutnjak et al. 2017; Arribas et al. 2018; Orton et al. 2020). Furthermore, the envelope of many animal viruses is built up from components of the host cell's membranes (Pratelli & Colao 2015; Perrier et al. 2019), as well as a capsid made of virus-specific proteins (Li 2016; Schoeman & Fielding 2019). To harden them against environmental offences and provide them with addressing tags, some of these proteins are glycosylated, which involves tapping into the cell's resources of UDP-sugars and GDP-sugar precursors (Wellen & Thompson 2012; Mayer et al. 2019). The very process of glycosylation via the endoplasmic reticulum requires dolichyl-phosphate, a terpene lipid unexpectedly phosphorylated by CTP-dependent dolichol kinase (Shridas & Waechter 2006). This further highlights the relevance of the membrane lipids, which uniquely derive from precursors involving pyrimidines, specifically liponucleotides based on a CDP skeleton (Kuo et al. 2016; Woods et al. 2016; Lee & Ridgway 2020). As a final key resource, we emphasize here the need for a virus to build up an active tRNA complement in order to express its proteins, necessitating the function of host tRNA-nucleotidyltransferase (CCAse). How the construction of the building blocks are put together in an uninfected cell is therefore the very first challenge faced by the virus after it has accessed the cytoplasm of the host cell. Finding an answer to this question dictates the exploration of the mystery of the cells' assembly lines that prepare them for growth. Before exploring phylogenetic consequences of this unique design, we propose in the next couple of paragraphs an integrated view of how cytosine-based metabolism is organized.

*The logic of energy management for nucleic acids synthesis*

Synthesis of the viral RNA genome draws resources not only from the metabolism of pyrimidines but also from the general logic of the cell's energy management. A key chemical feature of the related processes is that they rest on hydrolysis or synthesis of phosphate bonds (Westheimer 1987). Versatility of these processes is ensured by the usage of the shortest polyphosphate structure, that of nucleoside triphosphates, NTPs—we do not consider here the special case of purely mineral polyphosphates (Danchin 2009). RNA- and DNA-dependent genome synthesis is developed along two lines with respect to its energy demands (this is illustrated in **Figure 1** for pyrimidine metabolism).

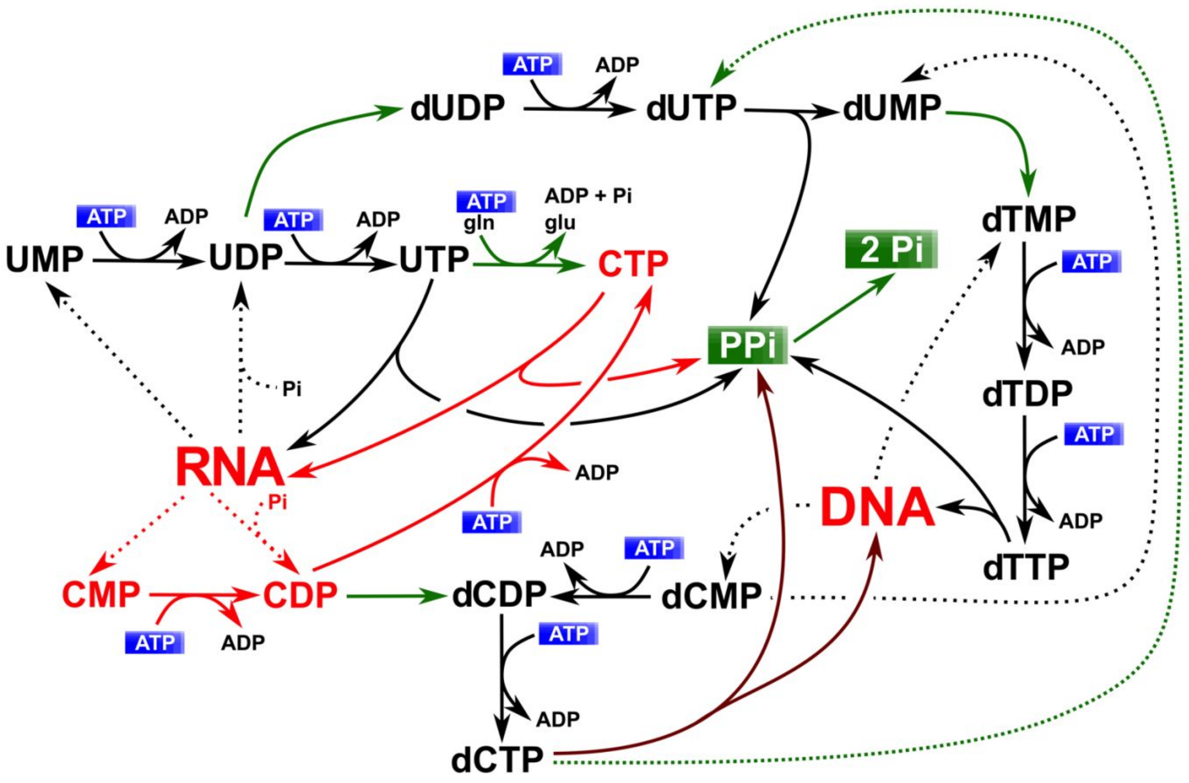

**Figure 1. Energy-driven pyrimidine-based nucleic acid metabolism**

ATP is the general donor in the biosynthesis of pyrimidines. CDP, required as a precursor of dCTP synthesis is produced by RNA turnover *via* hydrolysis or phosphorolysis (red arrows). RNA and DNA synthesis is driven by pyrophosphate hydrolysis (green arrows indicate irreversible reactions). dTTP results from a pyrophosphate-driven reaction producing dUMP, and is finely tuned by thymidylate kinase, which makes its immediate precursor dTDP.

When energy is meant to be used in a reversible way, NTPs are hydrolysed into NDP+Pi. This is where the role of mitochondria is critical, especially in non-proliferating cells (Maldonado & Lemasters 2014). These organelles restore the ATP complement of the cell, in particular to the endoplasmic reticulum—ER, (Yong et al. 2019), and in the present context this is crucial for the generation of new viral particles. In contrast—and this is relevant not only for intermediary metabolism but also for macromolecule biosynthesis, with more than 500 such reactions reported in the KEGG database (Kanehisa et al. 2017)—when the relevant pathways have to be driven forward, triphosphate hydrolysis produces pyrophosphate (PPi). PPi is subsequently hydrolysed irreversibly into two phosphates by omnipresent pyrophosphatases: NTP => NMP + PPi => NMP + 2 Pi, and this drives syntheses forward. Biosynthesis of macromolecules rests to a great extent on this two-pronged strategy. In parallel, the requirement of CDP for synthesis of the deoxyribonucleotide counterpart (**Figure 1**) limits the input of C nucleotides in DNA-based genomes (Rocha & Danchin 2002). Are RNA

viruses also submitted to patent metabolic constraints, and what would they be?

*An unexpected secret of life: cytosine metabolism provides both a rheostat and a flywheel to integrate growth of the various cell structures, constraining coronavirus development*

Surprisingly, the answer to this question is positive, with the involvement of cytosine nucleotides, again. All metabolites must be degraded and recycled, either as a whole or as parts. In the case of ribonucleotides, three units—a phosphate, a ribose, and a heterocyclic base—can go through specific degradation or salvage pathways. Strikingly, cytosine appears to sustain a privileged turnover metabolism, entirely poised to go via deamination to uracil, so that most of the cytosine nucleotide metabolism should go through phosphorylated forms, CMP and CDP for salvage, and CTP for *de novo* biosynthesis. Further in line with a general cytosine-based control, a specific pathway forms cytidine after hydrolysis of the 5'-phosphate of CMP, then deaminates it to uridine (Frances & Cordelier 2020), or, in bacteria but not in multicellular organisms, makes cytosine, which is subsequently deaminated to uracil (Ireton et al. 2002), then mainly scavenged directly by uracil phosphoribosyltransferase [UPRT, EC 2.4.2.9, **Figure 2** blue arrow] directly into UMP. That this indirect route plays a crucial role in cells is witnessed, for example, in the fact that the whole RNA-derived salvage pathway (cytidylate phosphatase and cytidine deaminase) is critical in embryonic development (Wegelin 1983). Furthermore, the very same enzymes of this salvage pathway are also important to recycle the modified derivatives of cytosine which result from frequent metabolic accidents or are encountered as epigenetic markers (Zauri et al. 2015) .

Thus, the salvage pathways are straightforward for all nucleobases, cytosine excepted (**Table 1**).

**Table 1. Cytosine-related salvage in human cells: present and missing enzymes with bacteria for comparison**

| | ***H. sapiens*** | ***E. coli*** | ***B. subtilis*** | **Enzyme name** |
|---|---|---|---|---|
| **Catabolism** | | | | |
| 3.5.4.12 | DCTD | absent | *comEB* | dCMP/CMP deaminase |
| 3.1.3.5 | NT5E<br>NT5C3<br>NT5C1A | *umpG*<br>*pynN(yjjG)* | *nucF(yutF)*<br>*pynN(yfnB)*<br>*ycsE*<br>*yktC* | 5'-nucleotidase |
| 3.5.4.5 | CDA | *cdd* | *cdd* | cytidine deaminase |
| 3.5.4.1 | absent | *codA (cda)* | absent | cytosine deaminase |
| 3.2.2.8 | absent | *rihA(ybeK)*<br>*rihB(yeiK)*<br>*rihC(yaaF)* | absent | ribosylpyrimidine nucleosidase |
| **Synthesis** | | | | |
| 6.3.4.2 | CTPS1<br>CTPS2 | *pyrG* | *pyrG* | CTP synthetase |
| **Salvage** | | | | |
| 2.7.4.14 | CMPK1<br>CMPK2 | absent | absent | UMP/CMP kinase |
| 2.7.4.25 | absent | *cmk* | *cmk* | cytidylate kinase |
| **Recovery from U** | | | | |
| 2.4.2.3 | UPP1 | *udp* | | uridine phosphorylase |

| | | | | |
|---|---|---|---|---|
| | UPP2 | | *pdp* | |
| 2.4.2.9 | UPRT | *upp* | *upp* | uracil phosphoribosyltransferase |
| 2.7.1.48 | UCK1<br>UCK2<br>URKL1 | *udk* | *udk* | uridine kinase |
| 2.7.4.22 | absent | *pyrH* | *pyrH* | uridylate kinase |
| 2.7.4.6 | NME1 – NME7 (7 genes) | *ndk* | *ndk* | nucleoside diphosphate kinase |

The purine salvage pathways have been thoroughly explored, in particular in animal pathogens and in plants (Ghérardi & Sarciron 2007; Ducati et al. 2011; Ashihara et al. 2018). By contrast, the pyrimidine salvage pathways have remained somewhat less studied (Villela et al. 2011). The omnipresent roles of ATP and S-adenosylmethionine (AdoMet) often ends up with adenine as a waste product. As a consequence, natural selection retained a variety of enzymes meant to scavenge adenine wastes, so that any downwards trend in the ever critical ATP supply would be easily overcome—see *e.g.* (Lüscher et al. 2014; Sekowska et al. 2019). A key enzyme in this salvage process is adenine phosphoribosyltransferase [EC 2.4.2.7, (Wilson et al. 1986), 1290 references in PubMed on 25/09/20]. In the same way, guanine (respectively, uracil) are salvaged *via* (hypoxanthine)-guanine phosphoribosyltransferase [EC 2.4.2.8, (Balendiran et al. 1999), 1545 references in PubMed on 25/09/20], [respectively UPRT, EC 2.4.2.9, (Li et al. 2007), 312 references in PubMed on 25/09/20]. Further comparable activities exist, such as orotate phosphoribosyltransferase EC 2.4.2.10 (Donini et al. 2017)—which inputs orotate at the beginning of the pyrimidine biosynthetic pathway—and the somewhat less similar nicotinamide phosphoribosyltransferase EC 2.4.2.12 (Grolla et al. 2020). These enzymes share a common descent, showing that life has easily evolved a panoply of related activities.

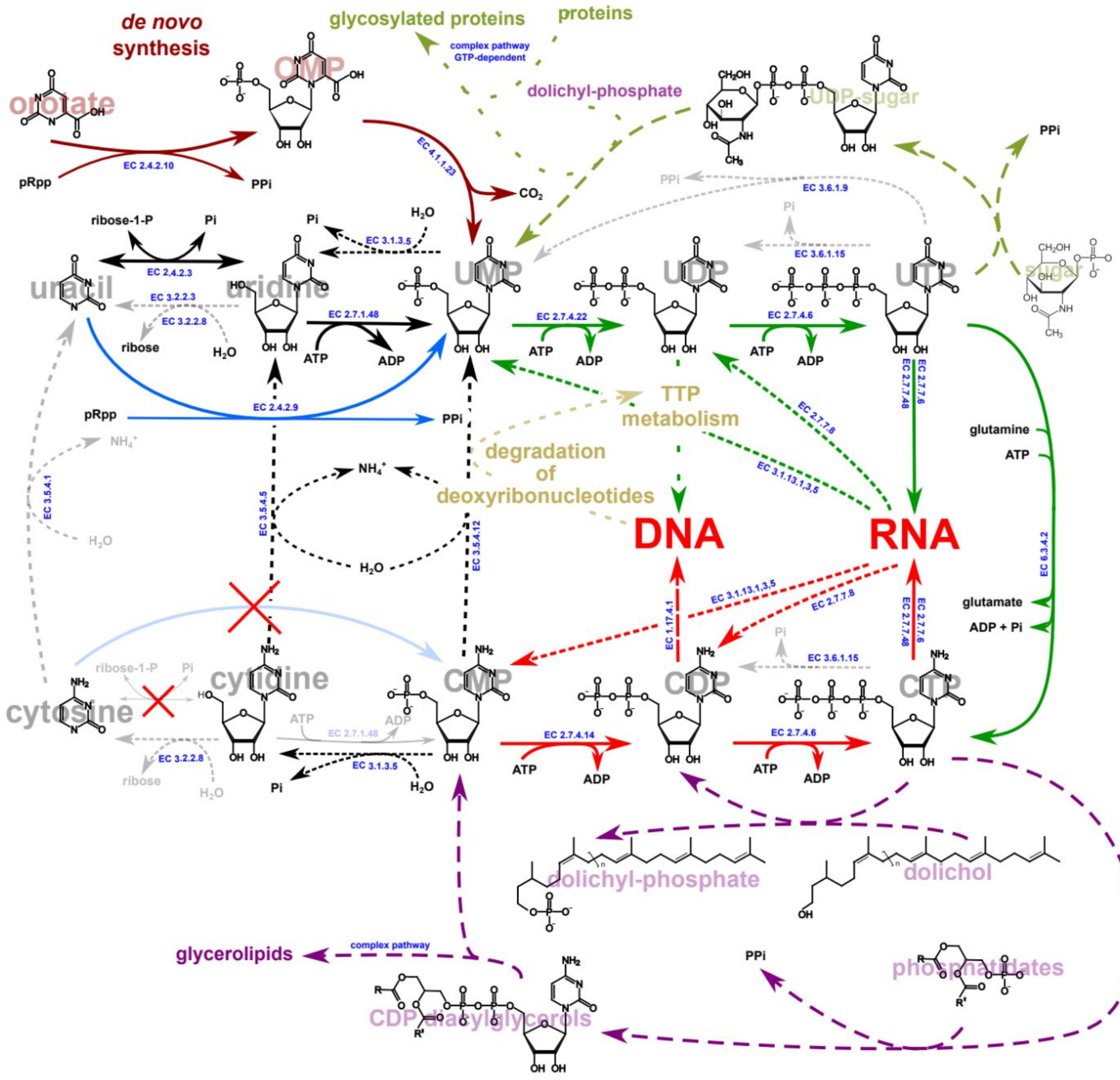

**Figure 2. Synthesis and salvage of pyrimidine nucleotides**

Synthesis of UMP begins with orotate phosphoribosyltransferase followed by decarboxylation (brown arrows). The anabolic pathway ends up with UTP and CTP (bright green arrows). Salvage of CTP stems from RNA metabolism (red arrows) and lipid metabolism (purple arrows). Degradation and scavenging of cytosine-based nucleotides goes through uracil-based scavenging and return to the CTP biosynthetic pathway, with cytosine deamination of intermediates as critical steps. UPRT matches the role of the orotate counterpart in the ultimate salvage of the base (blue arrow). No counterpart has been yet identified, to our knowledge (see text), for scavenging cytosine (crossed out light blue arrow). Distribution of relevant enzymes in *H. sapiens* and model bacteria is illustrated in **Table 1**.

It was therefore expected that the same would hold true for cytosine, allowing the cell to scavenge the base easily from its environment. Yet, we made the unexpected discovery that, to the best of our knowledge, no cytosine phosphoribosyltransferase exists in any extant organism (light blue arrow, **Figure 2**). This apparent deficiency might result from some moonlighting activity of UPRT allowing it to recognize cytosine. However this is unlikely. For example, in the minimal genome of *Mycoplasma mycoides* UPRT and cytidine 5'-triphosphate synthetase determine the rate of pyrimidine nucleotide synthesis, implying that there is no direct scavenging cytosine into CMP (Mitchell & Finch 1979). UPRT in *Escherichia coli* is highly specific for uracil and some uracil analogs (Rasmussen et al. 1986). The same is true in plants with a moonlighting enzyme that does not lead to CMP (Katahira & Ashihara 2002; Arrivault 2019), while mammals were supposed to lack this activity altogether (Cleary et al. 2005), until a structurally-related protein was identified, although failing to display UPRT activity (Ghosh et al. 2015). Finally the fact that pyrimidine metabolism flows essentially through uracil, not cytosine derivatives has been established in parasites (Dai et al. 1995; Schumacher et al. 1998). By contrast, early work with the protozoon *Giardia lamblia* suggested that this activity might be present in the organism (Aldritt et al. 1985; Jarroll et al. 1989). Surprisingly however, deciphering the genome strongly suggested that CTP was derived from cytosine deamination into uracil, followed by salvage of uracil and amidation. Indeed in the most recent release of GiardiaDB, there appears to be no sequence in the genome of the organism that could be attributed to cytosine phosphoribosyltransferase [see (Aurrecoechea et al. 2009) for access to the genome database]. At this point, therefore, no known extant organism codes for such an enzyme. By contrast, despite the lack of *de novo* biosynthesis pathways for pyrimidines in this organism, the genome still codes for a CTP synthetase (PyrG). This is in line with the general observation that cytosine and related cytosine-containing derivatives are systematically deaminated into uracil-containing derivatives, subsequently processed to regenerate CTP (**Figure 2**, and **Figure 1** for salvage of processed DNA derivatives). As a further case in point, *pyrG* has also been found as a necessary complement required for life in the smallest genome of an autonomous synthetic streamlined construct (Hutchison et al. 2016). This strongly suggests that recovering CTP requires a uracil-dependent pathway as well as that independent management of cytosine-based nucleotides is critical to govern metabolism, even in the presence of a rich supply of metabolites from the outside—note that *Giardia* is a parasite.

This singular positioning of CTP synthesis in metabolism makes CTP synthetase a convenient enzyme for the cell to adjust the flow of cytosine-containing nucleotides, acting as a rheostat does in an electric contraption—see *e.g.* (Shin et al. 2020). Substantiating this unique role, the functional structure of the enzyme displays a very unusual architecture. It makes filaments, named cytoophidia—specific membrane-less organelles that control the spatial distribution of cytosine-dependent intermediary metabolism (Liu 2010; Sun & Liu 2019)—in all the organisms where its organization has been explored (Li et al. 2018). The structure of CTP synthetase is important in the present context because the synthesis of membrane lipids is a further metabolic step that involves the nucleotide, with most membranes deriving from cytosine-based liponucleotides (Chauhan et al. 2016; McMaster 2018). Because the lipid content of cells can vary over a wide range, the stores of CDP-containing liponucleotides, in particular in eukaryotes—with an important network of intracellular membranes—is preset to play the role of a flywheel, allowing fine tuning of the availability of cytosine-derived metabolites in the cell when conditions vary. This property could have been advantageously recruited for innate immunity. Indeed, inactivation of one of the two human CTP synthetase genes strongly impaired lymphocyte function (Martin et al. 2020). Finally, as perhaps could be expected at this point of our demonstration, the very first enzymes for *de novo* synthesis of pyrimidines, carbamoyl-phosphate synthetase (*C*PSase), aspartate transcarbamylase (*A*TCase), and dihydroorotase (*D*HOase) are associated into a multifunctional structure, named CAD (Del Caño-Ochoa & Ramón-Maiques 2020). Besides a general role in management of cell growth, CAD is highly expressed in leukocytes, where it enables Toll-like receptor 8 expression in response to cytidine and single stranded RNA (Furusho et al. 2019), a situation met upon infection by RNA viruses. Supporting a role of CAD in antiviral innate immunity, its activity is modulated by a dedicated viral protein during Enteroviral infection (Cheng et al. 2020) .

As a matter of fact, most enveloped RNA viruses are low in C. When viruses of the same genus are vector-borne, they appear to display a different nucleotide composition, sometimes higher in G+C (Jenkins et al. 2001), indicative of some driving force due to the metabolism of the vector, not investigated at this time. There is also some specific examples of G+C-rich viral genomes such as that of the Rubella virus (Zhu et al. 2016). The reasons for this exceptional nucleotide composition is not known, and it has been attributed to inhibition of the APOBEC1-editing process (Khrustalev & Barkovsky 2011). However, such inhibition would require a specific viral function, a feature that we rather propose to see involved in modulating either CAD activity (as discussed above for enteroviruses) or preferably CTP synthetase. Our observations would therefore be extremely helpful in focusing research on identification of viral proteins interfering with cytoophidia.

**Consequences of cytosine-related imbalance in nucleotids composition, evolution and coding capacity of coronavirus genomes**

The most straightforward consequence of the metabolic qualitative design just outlined is that a metabolic force will keep driving the cytosine content of RNAs to lower values, unless opposite processes—and selection pressure leading to discard organisms with too low cytosine content, for example because this would create unbearable biases in the amino acid composition of the proteins coded by these genomes—had the upper hand during evolution. This prompted us to develop an explicit analysis of the consequences of pyrimidine metabolism’s organisation in relation with SARS-CoV-2 infection, as we now document.

*Cytosine content-related phylogeny of some virus isolates*

The constraint on cytosine availability witnessed in the composition of coronaviruses—in particular SARS-CoV-2—is likely to reflect the coupling between synthesis of viral particles and the host cell’s metabolic capacity. In order to assess the evolution of the virus with these metabolic constraints we evaluated the C content of their genome, using 89 representative strains from the four genera of coronaviruses, selected based on their phylogenetic and host background. Here we developed two distinct approaches in order to take into account 1/ the nucleotide patterns across the virus group, and 2/ the coding sequence-related limitations that constrain the function of the viral proteins as they adapt to their host.

To study the evolution of coronaviruses, we used standard techniques to generate a phylogenetic tree of representative strains (see **Materials and methods** section), where we highlighted the average cytosine content of each viral genome (**Supplementary Figure 1**). There is significant variation between the cytosine content of viruses of different clades. Based on 89 representative genomes we found that coronaviruses have 27.3% A, 17.9% C, 21.5% G and 33.3% U on average. Overall, the coronavirus genomes contain somewhat more pyrimidines than purines, with a mean content for C+U of approximately 51.2%.

Currently, seven coronaviruses are known to infect humans. These include four epidemic CoVs causing mild respiratory symptoms in humans (HCoV-229E, HCoV-NL63, HCoV-HKU1 and HCoV-OC43), the severe acute respiratory syndrome coronavirus (SARS-CoV-1) causing the pneumonia outbreak during 2002-2003 in China (Drosten et al. 2003), the Middle East respiratory syndrome coronavirus (MERS-CoV) still causing small outbreaks through camel-to-human transmissions in the Middle East and the newly emerged SARS-CoV-2 (Cui et al. 2019). These viruses all originated from bat species, although the exact intermediate hosts involved in human infections remains obscure (Corman et al. 2015; Huynh et al. 2012; Lau et al. 2015;

Moreno et al. 2017; Yang et al. 2016). Interestingly, the cytosine content of bat viruses is variable (from 16.0% to 21.5%), reflecting the diverse virus species harboured by bats, which make a very diverse natural reservoir for many coronaviruses. Intriguingly, we observed a lower cytosine content for the four human established epidemic viruses (HCoV-HKU1, 13.0%; HCoV-NL63, 14.4%; HCoV-OC43, 15.2%; HCoV-229E, 16.7%) than the three spillover-related viruses infecting humans (SARS-CoV, 20.0%; MERS-CoV, 20.3%; SARS-CoV-2, 18.4%) and most bat viruses. The four human epidemic viruses have been established in human populations for decades. The significantly low cytosine content observed for these two viruses may indicate host-related features impacting cytosine-containing metabolites availability. Several studies of the codon usage biases in emerging coronaviruses have been published, providing a general view of the situation—see e.g. (Tort et al. 2020), without, however, exploring the contextual constraints in terms of metabolic properties of their hosts.

If the adaptation of coronaviruses to the human species was to drive down the cytosine level in the virus genome, then we might expect the cytosine content of SARS-CoV-2 strains to decrease as the epidemic unfolds. Our regression model is consistent with this view (**Figure 3**).

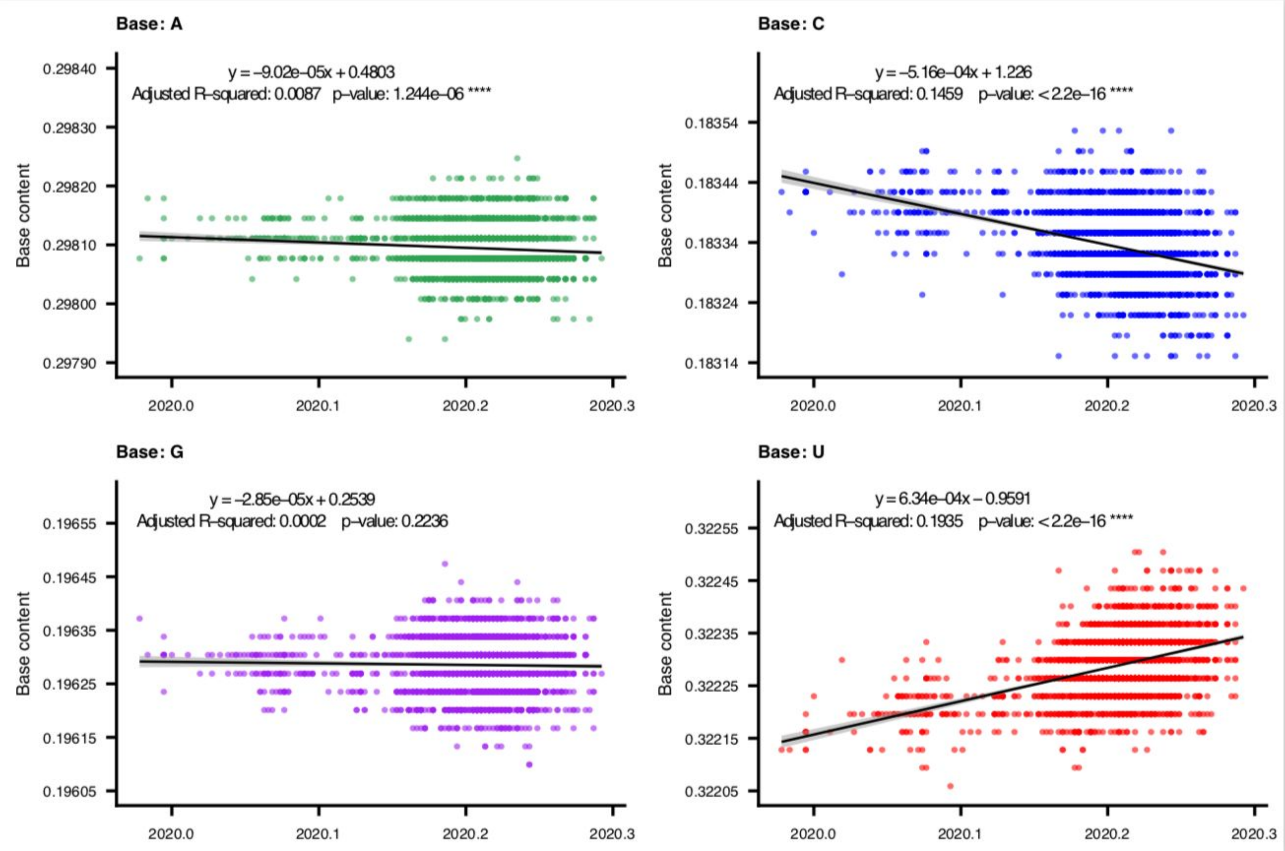

**Figure 3: Dynamic of base composition at the coding regions of SARS-CoV-2.**

The base composition of the coding regions concatenated by 26 ORFs of SARS-CoV-2 is displayed. Each dot represents one sequence. The calculation was based on 2,574 unique SARS-CoV-2 strains isolated from December 24$^{th}$, 2010 to April 17$^{th}$, 2020.

It allowed us to estimate that SARS-CoV-2 may lose its C complement by 0.000516 base per position per year (y = −0.000516x + 1.226, adjusted $R^2$ = 0.1459) while gaining U by 0.000634 per year (y = 0.000634x - 0.9591, adjusted $R^2$ = 0.1835), under its current circulation dynamics in susceptible populations. A parallel increased trend for A and a decrease for G was also observed, but with more moderate slopes than those for

C and U. A Wilcoxon rank sum test showed that the content of the four bases in the SARS-CoV-2 sequences was significantly different in each case, while somewhat correlated with each other. The strongest correlations were observed between two groups (**Supplementary Figure 2**): a decrease in A was significantly correlated with an increase in G (adjusted $R^2 = 0.5738$), while a decrease in C was significantly correlated with an increase in U (adjusted $R^2 = 0.7177$). As a consequence the SARS-CoV-2 virus is on its way to gradually lose C during its adaptation in humans, resulting in a genomic base composition more like those of the four previously established human endemic coronaviruses.

The second approach did not aim at creating a phylogeny of the viruses, but, rather, a cladistic tree showing structural properties shared by viruses likely to underlie functional features. This approach assumes that, after sufficient time of evolution, the nucleotides present at the majority of sites in the sequence had chances to be modified several times reaching local saturation, so that it is difficult or impossible to link those with specific features of the proteins encoded in the virus (the beginning and end of the sequence, critical for RNA-dependent replication are not taken into account). By contrast, the presence of insertions or deletions (indels) will affect considerably the overall structure of the proteins, and this should impact their function in a way that is not likely to be reversible—see for example (Zhou et al. 2020). An earlier such report (Sekowska et al. 2000) demonstrated the usefulness of this approach (Gupta 1998). The use of cladograms in this case attempts to show the relative distances and should not necessarily reflect the evolutionary history of the group.

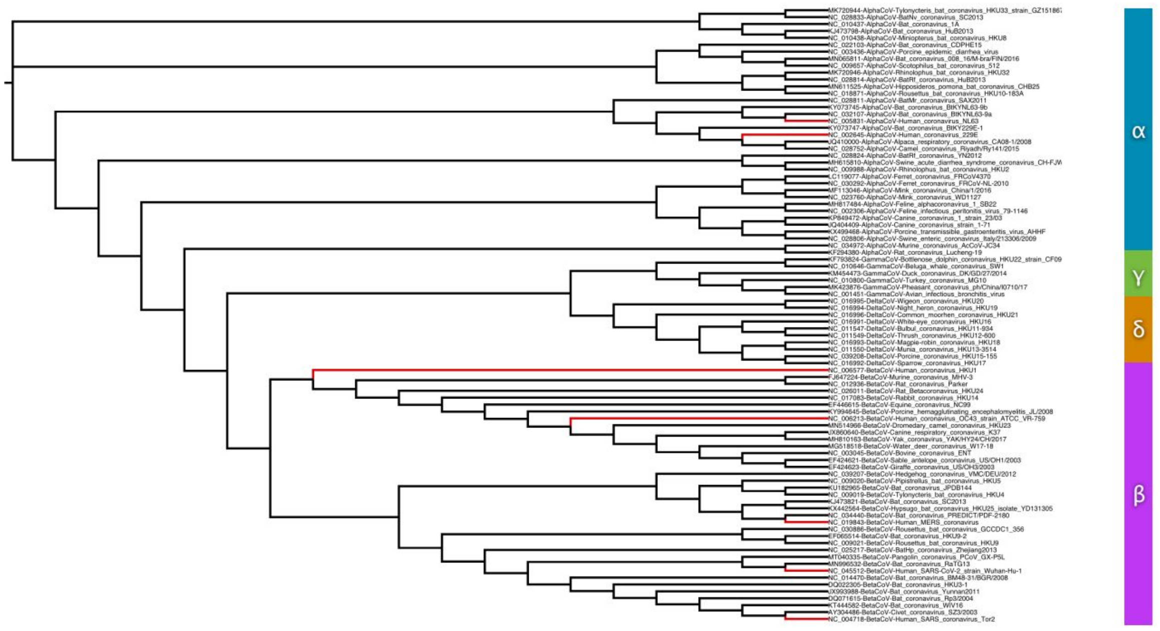

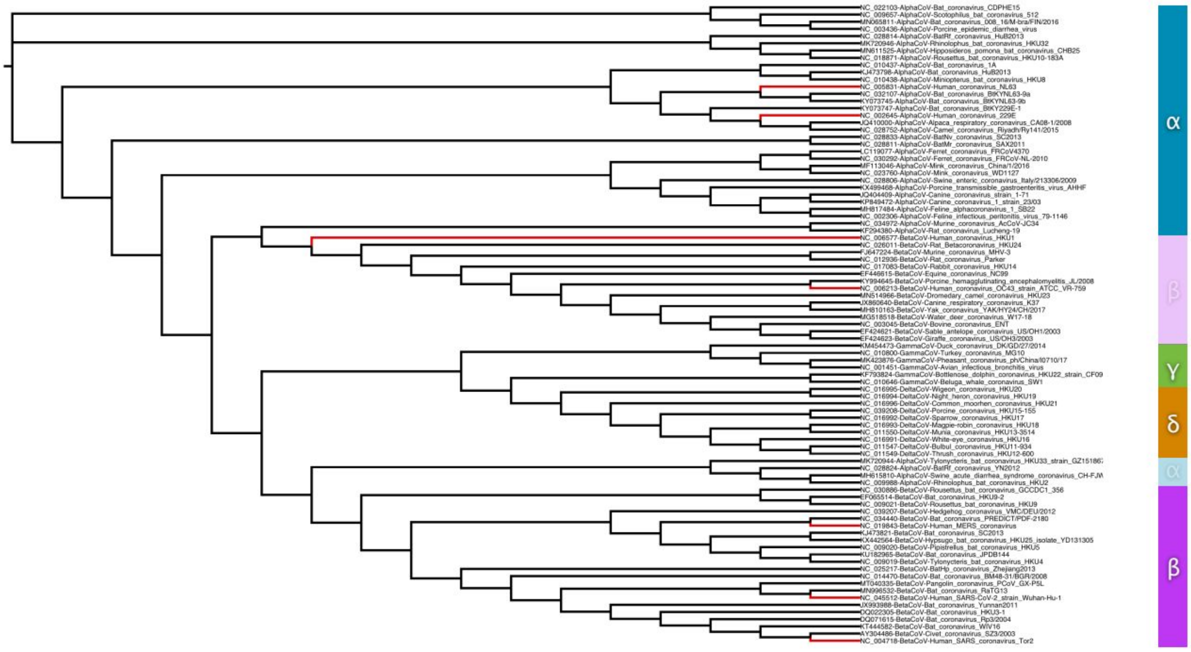

**Figure 4. Phylogeny of coronavirus representatives based on full genome sequences (upper panel) and on indels (lower panel)**
A genome-based tree is generated based on the full genome sequence alignment of the group (panel A) and a gap-based tree is created, based on insertions and deletions (indels) only (see Methods). The seven known human coronavirus strains are highlighted by a red colour for the corresponding branches. A panel on the right indicates the four coronavirus groups; in panel B, the two incongruent sub-groups are shown by the same colour code with reduced opacity (alpha and beta sub-groups). For details, please see text.

Compared to the standard alignment of the 89 reference genomes (**Supplementary Table S1** and **Supplementary Figure 1**), the equivalent gap-based alignment uses undefined characters for nucleotides and ‘dummy’ characters for gaps to cheat the algorithms for tree construction so that distances are calculated on the basis of the sums of scores for gap positions (presence of indels, see **Methods**). While it is not possible to check one by one each and every gap, the common ones are likely to reflect a common structure or function characteristic of the corresponding region (**Figure 4**).

Remarkably, both the standard, genome-based tree and the gap-based tree are quite congruent—i.e. knowing only the indel content is enough to draw a tree that describes the relationships between the coronavirus groups (**Figure 4**). In the genome-based tree, the four groups of coronaviruses are detected clearly, and the seven human virus strains are highlighted, in groups alpha and beta (**Figure 4a**). In the gap/indel-based tree, the four groups are also consistently derived, with the exception of a beta sub-group that contains the two human viruses with reduced pathogenicity potential, compared to the beta sub-group that contains the SARS, MERS and SARS-2 strains (**Figure 4b**). At the same time, a tiny alpha sub-group exhibits similar indel patterns with the latter beta sub-group, presumably with similar indel patterns. It is tempting to speculate that these alpha sub-group strains may share certain hitherto unknown properties that might render them potentially dangerous in terms of zoonotic disease capacity. The observed patterns could be interpreted as showing that what is coded in the indel regions has a considerable weight on the virus adaptation to their hosts and does not strictly depend on the base composition and amino acid coding potential of the genome sequences. More research is needed to establish the nature of indel-based trees in the future.

*Biased codon composition of the regions coding for individual viral proteins*

Coronaviruses, and many positive-sense single-stranded RNA viruses as well, produce plus strands at a 50- to 100-fold excess of their minus-strand replicated template. Because several regions in the 3' half of the virus are « transcribed » from the template RNA minus-strand of the virus (Yang & Leibowitz 2015), a further deviation from parity should appear in the nucleotide usage for virus construction. This means that the overall nucleotide consumption is not strictly constrained by the second Chargaff's parity rule, that would result in an amount of A equal to that of U, and G to that of C (Forsdyke & Mortimer 2000). The virus multiplication rests on a RNA-dependent replication process, so that any pressure on a given base availability—here C—would affect its complement—G in our case. As discussed in the previous section, we expected a general selection pressure operating on CTP and tending, in the long run, to decrease the C content of the RNA virus, but also that of G.

Furthermore, this implies a particular imbalance in the nucleotide composition of the viral RNA, allowing it to differ from standard mRNAs of the host cell. We therefore expect that the virus will interfere with the host's translation machinery in a way that allows it to be discriminated positively against the cell's mRNAs (see **Discussion**). This should have consequences for the translation of the viral genome. The virus encodes proteins that have essential functions for its development, not only for the replication machinery and the formation of a capsid, but also for several ancillary functions needed for hijacking the host metabolism. The constraint on the genome nucleotide composition must be reflected in the codon usage bias of the virus protein coding sequences, with important consequences on the way tRNAs are used. Furthermore because natural selection acts on viral functions, it can be expected that the outcome of the general C lowering trend will differ in different proteins encoded by the virus, depending on the selection pressure constraining their functions. Taking advantage of the degeneracy of the genetic code, SARS-CoV-2 could also limit its C content *via* the use of alternatives at the third codon position by selective codon usage.

To explore this hypothesis, the relative synonymous codon usage (RSCU) values were calculated for each coding region of SARS-CoV-2 to reveal any differential usage of synonymous codons. An RSCU value of 0, 0~0.6, 0.6-1.6 or >1.6 implies that a codon is not-used, under-represented, normally-used, or over-represented—respectively for the four value ranges (Uddin & Chakraborty 2017). Among the 26 major coding regions, six (Nsp11, ORF10, ORF7b, ORF6, E and Nsp7) have a translated peptide length of less than 100 amino acids, which would result in codon usage profiles of low statistical significance. For example, Nsp11 encodes only 33 amino acids, which explains the very limited number of codons utilized by the corresponding gene. These sequences were not further analysed (**Figure 5**). The three codon positions do not have the same importance in the selection of specific amino acids. Pressure against C must have consequences in terms of the protein coding landscape of the virus. In general, in regions where tRNA

choice allows a wobble between U and C, the virus sequence is considerably enriched in U. It seems noteworthy that the codon usage bias and bias in tRNA choice differs between genes of the human host and the virus genes, except for two viral proteins, accessory protein Nsp1 and nucleocapsid protein N, notwithstanding preference of U over C—e.g. preference for GGU and CGU codons in protein Nsp1 (**Supplementary Figure 3**).

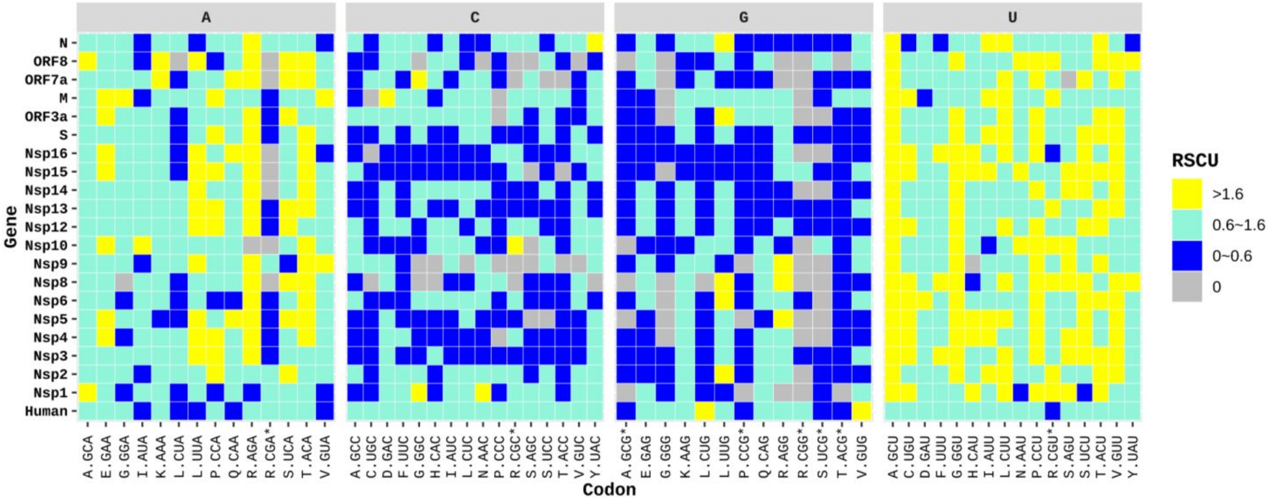

**Figure 5. Codon usage of SARS-CoV-2 ORFs based on the third codon position compared to human coding regions**

The 20 SARS-CoV-2 ORFs with a length of over 300 nucleotides were submitted to RSCU calculation. The codon usage for 120,426 human coding regions was also displayed to facilitate comparison. Codons are displayed with the first letter denoting the amino acid and the three letters following a dot representing the codon. Codons containing a CpG dinucleotide are suffixed by an asterisk. The four panels separate the codons according to the nucleotide located at the third codon position. Codons that are not used (RSCU=0), under-represented (RSCU<0.6), normally utilized (RSCU ranges between 0.6-1.6), or over-represented (RSCU>1.6) are labeled in grey, blue, ice cold and yellow, respectively.

The first C codon position is used to input histidine, glutamine, proline, and arginine or leucine in proteins. This is particularly significant for the proline residue, essential in the folding of key viral protein domains (Li *et al.*, 2014), because it is encoded by CCN codons. All proteins of SARS-CoV-2 prefer the usage of CCA or CCU codons for proline, avoiding the usage of C and G (**Figure 5**). Histidine and glutamine are in two-codon boxes, discussed below. Arginine presents a different situation because CGN codons can be replaced by AGR codons: SARS-CoV-2 favours the AGA codon especially, with only one compulsory G. AGG codons are enriched in proteins Nsp5, Nsp8 and Nsp9. Remarkably however, the Nsp1 protein, which corresponds to the initial domain of the ORF1a(b) protein, and is translated very early on in the virus expression cycle, contains only CGH codons (H = A, U or C) and this is in total contrast with the other viral proteins (except for Nsp10, yet this protein has only two arginine residues, making this observation possibly irrelevant). Codon CGG is only present 11 times in the coding sequences of the virus, suggesting that when present, it has been submitted to positive selection, possibly at a site important for the translation-coupled folding of the protein. The most interesting location of this codon is a doublet that corresponds to a four codon insertion in the spike protein of the virus. Finally, the pressure on leucine content is also lower. CUN codons are used to code for leucine, with the majority using codon CUU, but this amino acid can be introduced using the alternative UUR codons. Yet, UUA is used more frequently than UUG. UUG is relatively enriched in proteins Nsp6, Nsp8, ORF3a and N (**Figure 5** and **Supplementary Figure 3**).

In the second position requiring a C we find proline again, and also threonine (ACN), alanine (GCN) and serine (UCN). For threonine, codon ACU is the most used codon, progressively being generally replaced by ACA as we progress along the genome sequence, ACC and ACG are rare. Alanine is mainly encoded by GCU codons, followed by GCA, with protein Nsp1, again, differing somewhat from the other viral proteins in that the frequency of GCA and GCU are the same. Serine (UCN) is able to escape much of the constraint imposed by C availability as it can use the alternative AGY codons. Codons UCU and UCA are more or less used in an equivalent way, except, again, in protein Nsp1, which mainly uses AGU and AGC codons. In general AGC is seldom used while AGU is the dominant serine codon.

Finally, the third position can be replaced by A, U or G in the four codon boxes, two of which, valine and glycine, are further discussed below. In general the corresponding NNC codons are rarely used. Again, protein Nsp1 is an exception, with codon GGC used more frequently than GGU. Overall GGU is dominating with some contribution of GGA, while GGG is often absent. In the case of valine (GUN codons), the dominating codon is GUU, followed by GUA. By contrast, U-ending codons which are rarer than expected are clustered in several proteins: UGU, UUU and UAU in protein N; AUU in Nsp10; CGU in Nsp16; GAU in protein M, CAU in ORF8 and AAU and UCU in Nsp1. NAU codons correspond to two codon boxes (NAN codons). These codons are discriminated along a pyrimidine / purine axis. A pyrimidine (NAY) is used to maintain the same nature of the coded residue whether the codon uses a U or a C as its 3' end (aspartate, asparagine, histidine and tyrosine), while a purine (NAR) allows coding for glutamate, glutamine and lysine. UAR codons are also specifying the terminal step of translation. As stated above, the SARS-CoV-2 genes avoid the usage of C containing codons whenever possible (**Figure 5**). Moreover, probably due to the base-pairing requirement imposed during transcription and replication, the virus also avoids the usage of G-ending codons. This avoidance is maintained in the overall choice of NAR codons, except in protein Nsp6 where CAG is preferred to CAA, as well as GAG over GAA and CAA over CAG, which suggests that this results from a significant selection pressure. This is the more remarkable because Nsp proteins are cleaved off large ORF1a and ORF1ab precursors. In general, and this is as expected, codons NAU are preferred over NAC for the pyrimidine ending codons of NAN boxes. The exceptions are, for GAC, protein Nsp5 and protein M; for CAC, Nsp10 and ORF7a; for AAC, protein Nsp1 and protein M; and for UAC, proteins Nsp1, Nsp5, Nsp9, M, Orf7a and N.

### *tRNA-dependent modulation of synonymous codon translation*

Consistent with metabolic pressure against CTP and despite their uneven coding length, most of the genes of SARS-CoV-2 avoid the usage of the C-ending codons (**Figure 5** and **Supplementary Figure 3**). A similar low preference for C-ending codons was also observed for coronaviruses of other species and genera, as calculated using the ORF1ab coding region. The way tRNAs are utilized as a function of the first anticodon (third codon) nucleotide is highly unsymmetrical. For this reason the corresponding tRNA position (N34) is usually heavily modified, while specific tRNAs are deciphering individual codons (**Table 2**). Interestingly, this constraint is easily matched by the tRNA supply of the cell because, contrary to codons ending with a purine, which require distinct tRNAs to be decoded, codons ending with a pyrimidine (U or C, Y) are sometimes decoded by a common tRNA species. For NAY codons, the position 34 of tRNAs is a G, replaced by queuine (Q), if this metabolite of bacterial origin is present in the host. Availability of Q in the environment may not have major consequences for translation of NAC codons, but it may alter the speed and accuracy of translation of the NAU codons. G-ending codons are generally rare in the virus, but they do not systematically correspond to rare tRNAs (**Table 2**). Because the anticodon position 34 of the cognate tRNAs is either a guanine or a queuine (Q) residue (depending on a specific input from the environment) and because NAU codons are translated in the absence of Q more slowly and less accurately than NAC codons, a pressure towards NAC in a context where C availability seems to be limiting is probably significant. This may apply to protein Nsp1 and to a lesser extent to protein M (see **Discussion**). All transfer RNA (tRNA) decoding strategies depend on the type and extent of modifications at position 34 of the tRNA anticodon (Grosjean & Westhof 2016). The codon usage bias in SARS-CoV-2 differs from that of average human proteins. In particular it is enriched in codons that require tRNAs modified at position N34 of the anticodon with complex modifications that are linked to zinc homeostasis (Danchin et al. 2020), an important feature knowing that several of the virus functions are $Zn^{2+}$-dependent, while antiviral protein ZAP is a zinc-finger protein (Meagher et al. 2019).

**Table 2. Table of the genetic code with emphasis on decoding by individual human tRNAs**

| | A | | G | | U | | C | | |
|---|---|---|---|---|---|---|---|---|---|
| A | Gm (10) | Phe | I (9) | Ser | G/Q (13*) | Tyr | G [C32 mod Ψ, 29] | Cys | A |
| G | | | | | | | | | |
| U | xcm$^5$U (4) | Leu | ncm$^5$U (4) | | | ter | | ter | |
| C | C (1+5*) | | C (4) | | | ter | Cm (7) | Trp | |
| A | I (9) | Leu | I (9) | Pro | G/Q (10) | His | I (7) | Arg | G |
| G | | | | | | | | | |
| U | U (3) | | ncm$^5$U (7) | | xcm$^5$s$^2$U (6) | Gln | mcm$^5$U (6) | | |
| C | C (9) | | C (4) | | C (13) | | C (4) | | |
| A | A (14) | Ile | I (9) | Thr | G/Q (20) | Asn | G (6) | Ser | U |
| G | G (3) | | | | | | | | |
| U | Ψ (5*) | | ncm$^5$U (6) | | xcm$^5$s$^2$U (12) | Lys | xcm$^5$U (1+5*) | Arg | |
| C | C (9+1) | Met | C (5) | | C(15) | | C (5) | | |
| A | I (9) | Val | I (22) | Ala | G/Q (13) | Asp | G (14) | Gly | C |
| G | | | | | | | | | |
| U | ncm$^5$U (5) | | ncm$^5$U (8) | | xcm$^5$s$^2$U (7) | Glu | xcm$^5$Um (9) | | |
| C | C (11) | | C (4) | | C (8) | | C (5) | | |

The table displays the distribution of the 415 tRNAs coded in the human genome, among which 28 (noted with an asterisk) must splice out an intron as a maturation step: 5 $tRNA_{Arg}$ (decoding AGA), 5 $tRNA_{Leu}$ (decoding UUG), 5 $tRNA_{Ile}$ (decoding AUA) and 13 $tRNA_{Tyr}$ (decoding UAC/UAU). Indication of modifications is provisional as many modifications are not yet biochemically identified in human cells (de Crécy-Lagard et al. 2019).

A prominent feature is that two A-ending codons, CUA and CGA are generally rare in the virus sequence (**Figure 5** and **Supplementary Figure 3**). This is significant, as witnessed by the facts that the cognate codons CUU and CGU are particularly frequent (remember that A and U are both abundant in the virus genome). CUA is decoded by a specific $tRNA_{Leu}$ which is subject to specific regulation (Frias et al. 2013). An exception is, as reported above, the AGA codon, which is particularly frequent, and this makes protein Nsp1 stand out, highlighting further A-ending codon deficiencies (GGA, CUA, CCA, AGA and GUA). The deficiency of A-ending codons for nucleocapsid protein N, which is very rich in arginine residues and has the expected excess of AGA is limited to UUA, AUA, CUA and GUA, which corresponds to a UpA deficiency in mammalian genomes (Belalov & Lukashev 2013).

While the virus must certainly manage tRNA availability and adapt this resource to its specific codon usage bias, it must also curb the innate antiviral immunity which affects the pool of tRNAs directly. In human cells, tRNA molecules are synthesised as precursors that are maturated into pre-tRNAs that lack their CCA terminal end and need to be further modified (Slade et al. 2020). Remarkably, stress-induced synthesis of the specific protease angiogenin removes these CCA termini, stopping translation (Czech et al. 2013). Cells can overcome this process using tRNA nucleotidyltransferase and CTP and ATP. This is yet another CTP-controlled function that must be overcome by the virus. The specificity of angiogenin is modulated by tRNA modifications—this protease can also cut the tRNA molecules at sites located in their anticodons (Su et al. 2019)—and this may create an uneven selection pressure on the various tRNAs used to decode the virus genes.

**DISCUSSION**

All viruses must tap into their host resources to build up multiple copies of their genome and their envelope. In the present study we have documented the key role of CTP as a general coordinator of the cell's metabolism. This has unique consequences for the replication and evolution of enveloped RNA viruses, coronaviruses in particular. Metabolic availability of this nucleotide drives synthesis of the viral genome, its envelope, maintains the translation machinery and controls protein glycosylation. This coordinated role makes us understand the presence of the general innate immunity antiviral metabolite, 3'-deoxy-3',4'-didehydro-CTP (ddhCTP), produced from CTP by the interferon-induced protein viperin (Ebrahimi, Howie, et al. 2020; Gizzi et al. 2018). A general role of this unexpected metabolite has even been established in a work published during revision of this manuscript as important in the fight of prokaryotes against their phages (Bernheim et al. 2020). Indeed the role of ddhCTP has long been elusive, with experiments suggesting interference with RNA replication/transcription (Ng & Hiscox 2018), while others demonstrated interference with lipid metabolism (Nelp et al. 2017). It has also been shown that ddhCTP affects general metabolism via inhibition of $NAD^+$-dependent enzyme including the housekeeping enzyme glyceraldehyde-3-phosphate dehydrogenase (Ebrahimi, Vowles, et al. 2020). In this respect it seems revealing that *E. coli* CTP synthase is inhibited by NADH and other nicotinamides (Habrian et al. 2016). Cytoophidia have been observed to associate with IMP dehydrogenase to coordinate nucleotide metabolism (Chang et al. 2018; McCluskey & Bearne 2018). providing still another link between NAD-dependent enzymes and the multiplication of coronaviruses. No work, at this time, has shown that it should impact tRNA synthesis via inhibition of CCAse, a further action of this analog of CTP. How did this integrative role of CTP emerge during evolution?

Cells do not have to grow during viral infection. Yet, they result from billion years of evolution based on growth. The fate of the virus might therefore differ widely if the cells belong to classes that are normally poised to grow if triggered by relevant signals, or cells that are not meant to grow (such as neurons or cardiomyocytes). Accounting for growth in a three-dimensional space—the physical space where material entities such as most cells flourish—this literally asks for squaring the circle because growth of the cytoplasm (three dimensions) must be matched with growth of the membrane (two dimensions) and growth of the genome (one dimension). Putting together these three facets while sharing a common metabolism cannot be straightforward. Alas, as often in biology, solving a clear functional problem results more often than not in *ad hoc* solutions built on a fairly haphazard collection of bits and pieces, with considerable differences between different species. This would then preclude any consistent view of the anecdotes invented during evolution and end up in a catalog of solutions, as witnessed in the millions of articles that tackle biological questions. We could anticipate that the extensive evolutionary time scale allowed for a slow progression, exploring an infinite variety of directions.

Yet, we could be—and have been—lucky, as we discovered a universal set-up that may have some generality or even span the whole tree of life solving the growth hurdle. Because the number of the cell's building blocks is small (mainly nucleotides, amino-acids, phospholipids and carbohydrates), natural selection did recruit a limited number of those components to implement homeostatic regulation of the growth of the various cell compartments. From detailed analysis of the genome signatures of various organisms and their metabolic constraints, we have demonstrated here that the biosynthetic and salvage pathways leading to CTP had remarkable consequences in organising core cellular functions. A first pointer to this discovery was presented in (Danchin & Marlière 2020) and a detailed view is now presented in **Figure 2**. The highly involved set-up of this metabolic facet is significant for the manner and process by which a virus invades a cell and subsequently evolves a progressively better adapted progeny. Here we explored, using a functional analysis approach, how understanding this exceptional setup of intermediary metabolism allowed us to anticipate this particular aspect of the evolution of RNA viruses. The main conclusion we reached is that, overall, the coronavirus genomes had a tendency to shed their cytosine—respectively guanine—complement, essentially replacing it by uracil—and to a lesser extent guanine by adenine.

While this tendency constrains the genome as a whole, it is obvious that this will dramatically restrict the evolutionary trajectory of the virus, presumably leading to attenuation in the long terrm. Evolution however systematically uncovers negative counterparts for each novel function. This implies that the CTP-related armour defect constraining viral multiplication could be antagonized by specific viral functions, inactivating the interferon response or possibly specifically modulating the activity of CTP synthetase. This should be explored by metabolically focused studies of C-enrichment in some RNA viruses, such as that of the non-enveloped hepatitis E virus (Bouquet et al. 2012). In the case of SARS-CoV-2 and in the short term, because a major component of the antiviral innate immunity results from the production of the CTP analog ddhCTP, losing C residues in the genome will transiently alleviate some of the negative pressure created by this antiviral response. This also will help the virus to escape the limited, because it is highly context-dependent, deamination by APOBEC proteins (Milewska et al. 2018). A negative consequence of this genome composition trend might somehow account for the increase in virulence when a fairly GC-rich virus of an animal comes to infect a foreign host. Furthermore, occasional C-enrichment, resulting from inevitable template misreading, may be stabilized if the function of the corresponding translated polypeptide contributes to the production of a larger progeny of the virus. This makes identification of the functions associated to loci that do not readily comply with the loss of C (and G) residues as likely candidates important for a stable virus evolutionary potential.

Immediately upon internalization of the virus, its 3'-capped RNA genome begins to be translated into two large proteins coded from ORF1a and ORF1ab which contain a protease domain that cuts off 16 accessory proteins required for specific functions of the virus (Lu Wang et al. 2020). Its N-terminal domain, processed into non structural protein Nsp1, immediately interferes with translation of the host proteins—it is also inhibiting its own translation thus producing homeostatic regulation—by blocking the assembly of ribosomes that are in the process of translating host mRNAs and disrupting nuclear-cytoplasmic transport (Gomez et al. 2019). Subsequently a large complex forms with all the other Nsp proteins generated from the processed precursor, generating a RNA-dependent replication/transcription complex (RTC). Remarkably, this complex is tightly linked to key elements of the translation machinery. It has been demonstrated that, besides inhibition of interferon signalling, Nsp1 binds to the 40S ribosomal subunit (Kamitani et al. 2009) and that it further triggers host mRNA degradation (Narayanan et al. 2015). Nsp1 binds translation factors eIF3, eIF1A, eIF1 and eIF2-tRNAi-GTP (Thoms et al. 2020) and inhibits formation of the translation initiation complex—48S complex and formation of active 80S ribosomes (Lokugamage et al. 2012). Early interaction with the host translation machinery will stop translation, triggering host mRNA decay, which both produces nucleotide precursors for replication of the virus and hijacks the machinery to perform further translation of the viral genome. This implies that the complex between Nsp1 and the translation initiation complex is able to discriminate between different classes of mRNAs to allow or prevent their translation. Among the factors bound to Nsp1 the enigmatic ATP-dependent enzyme ABCE1 has been identified (Thoms et al. 2020). Remarkably, this protein is expected to behave as a “Maxwell’s demon” as do proteins of the EttA family in Bacteria, allowing partition of specific mRNA families the expression of which needs to be co-expressed or co-repressed (Boel et al. 2019).

This role of translation is apparent in the codon usage bias of Nsp1, which differs from that of subsequent domains cleaved off ORF1a and ORF1ab polypeptides. Here the role of arginine residue codons seems to have been submitted to strong selection, with a majority being CGU codons, while AGA and AGG codons—AGA translation being over-represented in the subsequent polypeptides—are totally absent from the sequence. Also, the arginine codon CGG is extremely rare overall in the genome sequence, and its locations are revealing, likely to be important for the co-translational folding of cognate proteins. This is particularly important in SARS-CoV-2 as the insertion generating its furin-like cleavage site in the spike protein that mediates cell entry (Follis et al. 2006; Belouzard et al. 2009; Coutard et al. 2020) is located right at a CGG doublet. Besides protein Nsp1, we demonstrated that the nucleocapsid protein N had also a general distribution of the codon usage bias that differed from that of the bulk of proteins coded from the ORF1a and ORF1ab regions. This is likely to be due to the fact that the corresponding transcripts also code for another protein in a different reading frame, protein ORF9b (Shi et al. 2014), and we can assume that this observations substantiates that this protein has indeed an important role in the biology of the virus. Remarkably, this also makes that the codon and tRNA usage bias of protein N resembles that of the human host. Whether this is meaningful should be further explored.

## PERSPECTIVES

Here, we reviewed the role of a specific intracellular metabolic pressure that must constrain the evolution of the genome sequence of RNA viruses, with emphasis on SARS-CoV-2 and in the context of the entire coronavirus family. Several studies have noticed the cytosine deficiency in the genome of evolving coronaviruses, with concomitant deficiency in the position of codons, but these observations were ascribed to deamination of cytosine resulting from the action of the host APOBEC system (Milewska et al. 2018) or to methylation of CpG dinucleotides (Yong Wang et al. 2020) as driving forces for evolution. By contrast, our working hypothesis is that the availability of CTP (and hence of cytosine-based precursors) is a dominating driving force in the way the virus evolves a new progeny. We are well aware that, due to the small number of samples and fairly short life time (as compared to usual evolutionary trajectory of a virus species), sequence analyses would provide only a limited view of sequence evolution and should be used more as a “rule of thumb” than a view based on trustworthy statistics. However we believe that, in view of the urgent situation we are facing, it is important to communicate our observations while relating them to previously unrecognised pressure that must have considerable importance in the evolution of viruses and the metabolic backdrop of its biology in the host cell. In this respect it seems worthwhile to explore whether unappreciated functions coded by viruses will be involved in controlling CTP availability.

## MATERIALS AND METHODS

### Data preparation

Dataset of reference coronaviruses: viral genomes were downloaded from GenBank (https://www.ncbi.nlm.nih.gov/genbank/) and GISAID (https://www.gisaid.org/). Representative coronaviruses of different species were selected from complete genomes, with reference genomes recommended by the Coronaviridae Study Group of the International Committee on Taxonomy of Viruses (https://talk.ictvonline.org/) and NCBI retained preferentially. For viruses containing isolates from different hosts, at least one representative strain from each host was kept. The genomes were aligned using MAFFT v7.427 (Katoh et al. 2002) and manually checked with BioEdit. Alignment of full genomic sequences were used for phylogeny reconstruction, while the coding regions for ORF1ab were extracted for codon usage analysis.

Dataset of SARS-CoV-2: a total of 17037 SARS-CoV-2 related sequences were available from GISAID on May 6$^{th}$, 2020 (Elbe & Buckland-Merrett 2017). Only SARS-CoV-2 genomes isolated from human, with a full length over 27,000 bp, no ambiguous sites and detailed collection date information were used for alignment. For duplicate sequences, only the earliest isolate was kept. Sequences for 26 coding regions, including Nsp1, Nsp2, Nsp3, Nsp4, Nsp5, Nsp6, Nsp7, Nsp8, Nsp9, Nsp10, Nsp11, Nsp12, Nsp13, Nsp14, Nsp15, Nsp16, S, ORF3a, E, M, ORF6, ORF7a, ORF7b, ORF8, N and ORF10 were extracted for each strain, using NC_045512 as reference. The coding sequences were checked manually to exclude those with abnormal mutations and early stop codons. A total of 4,110 strains with all 26 coding regions of complete ORF length were retained. After further deduplication based on the concatenated sequences comprised of the 26 ORFs, the final dataset contained a total of 2,574 unique SARS-CoV-2 isolates.

### Phylogeny reconstruction

Phylogenetic tree of the 89 representative coronaviruses was inferred using the Maximum Likelihood (ML) method implemented in IQ-TREE v1.6.12 with the GTR+F+I+G4 substitution model determined by

ModelFinder (Nguyen et al. 2015; Kalyaanamoorthy et al. 2017; Hoang et al. 2018). Ultra-fast bootstrap support values were calculated from 1,000 pseudo-replicate trees (Kalyaanamoorthy et al. 2017). Visualization of phylogenies were conducted with ggtree package (Yu 2020).

**Gap-based alignment**

The full alignment of the 89 reference strains was used to generate a tree, using FastTree 2.1.10 (Price et al. 2010) (with gamma distribution and the nucleotide option on – namely with the command options -gamma -nt), on the NGPhylogeny.fr server (Lemoine et al. 2019). The Jukes-Cantor model with balanced support Shimodaira-Hasegawa test was selected (Shimodaira & Hasegawa 1999). Total branch length was: 14.267.

Furthermore, a gap-based alignment was created, using gaps as follows: all dinucleotides were replaced with the 'undefined' symbol 'x' and the 'dummy' symbols (W for 3, Y for 6 and F for 9 consecutive gaps and the V symbol for all single gaps), leaving only single-nucleotides in-between gaps as anchor points (7% of total). The encoding in gaps of 3/6/9 are use to emulate the importance of potential codon gaps (reflected in the BLOSUM45 matrix). Total branch length was: 1.673.

Gap-based genome-based phylogenetic reconstruction for this group is based on the fact that, as also mentioned recently elsewhere (Li et al. 2020), these viruses undergo significant recombination and a large number of nucleotide positions achieve saturation thus confounding phylogenetic signal. Tree visualization was facilitated by IcyTree (Vaughan 2017)

**Base content calculation**

Base content was calculated by dividing the occurrence of each base by the total length of the sequence. Genomic base contents of representative coronaviruses were calculated with the full viral genome sequences. For the base content dynamic analysis of SARS-CoV-2, base compositions were calculated using the 2754 unique sequences concatenated by 26 ORFs.

**Codon usage analysis**

Codon usage analysis was conducted based on the ORF1ab region of representative coronaviruses and the 26 individual ORFs of SARS-CoV-2 strains. Relative synonymous codon usage (RSCU) value was defined as the ratio of the observed codon usage to the expected value (Sharp & Li 1986). Codons with an RSCU value of 0, 0~0.6, 0.6~1.6 or > 1.6 were regarded as not-used, under-represented, normally-used, or over-represented (Uddin 2017). RSCUs for the 120,426 human coding regions were determined based on the *Homo sapiens* codon usage table retrieved on 2020 June 14$^{th}$ from TissueCoCoPUTs (Kames et al. 2020).

**Statistical analysis and plots**

Statistical test, linear regression and data visualization were all conducted in R. Kruskal-Wallis test by rank and Wilcoxon rank sum test for pairwise comparisons were applied as appropriate. P values are labeled as follows: < 0.0001, ****; 0.0001 to 0.001, ***; 0.001 to 0.01, **; 0.01 to 0.05, *; ≥ 0.05, not labeled. $p<0.05$ was considered as significant.

**ACKNOWLEDGEMENTS AND FUNDING INFORMATION**

This work was supported by Stellate Therapeutics and the National Science and Technology Major Project of China (No. 2017ZX10303406), the emergency grants for prevention and control of SARS-CoV-2 of Ministry of Science and Technology (2020YFC0841400) and Guangdong province, China (2020B111107001, 2020B111108001). We warmly thank Prof. Huanming Yang, Dr. Ziqing Deng and Dr. Minfeng Xiao for their constructive communication in study design and data interpretation. Our thanks also go to Mr. Jielun Cai and Miss Xinyi Cheng for their assistance in data preparation. Thanks also to Pierre-Yves Bourguignon for his analysis of the cytosine complement of the virus and to Agnieszka Sekowska for her comments on metabolic pathways. We thank all the authors who shared genomic data in public database, and an acknowledgment table for sequences retrieved from GISAID (Elbe & Buckland-Merrett 2017) is provided (**Supplementary Table S2**).

**CONTRIBUTIONS**

AD designed the study and wrote the bulk of the manuscript. ZO, JL and WC developed the in silico analyses of cytosine evolution, codon usage biases and phylogeny. ZO, DW and WS performed the analysis and ZO wrote the corresponding part of the manuscript. CO designed and performed phylogenetic studies based on indels. PM identified several steps of CTP metabolism critical for virus proliferation. All authors read, wrote some sections and contributed to the final version of the manuscript.

**CONFLICTS OF INTEREST**

AD is a founder of Stellate Therapeutics, a company developing applications of metabolism for prevention and cure of neurodegenerative diseases and a founder of Virtexx, a company developing antiviral molecules. ZO and authors from Shenzhen are employed by the BGI, a company developing applications of genome studies. PM is a founder of Theraxen, a company developing synthetic biology approaches for drug development. The other authors declare no conflict of interest.

**ARXIV**

A first version of this article has been deposited at the bioRxiv repository under reference https://biorxiv.org/cgi/content/short/2020.06.20.162933v1

**FIGURE LEGENDS**

**Figure 1. Energy-driven pyrimidine-based nucleic acid metabolism**

ATP is the general donor in the biosynthesis of pyrimidines. CDP, required as a precursor of dCTP synthesis is produced by RNA turnover *via* hydrolysis or phosphorolysis (red arrows). RNA and DNA synthesis is driven by pyrophosphate hydrolysis (green arrows indicate irreversible reactions). dTTP is results from a pyrophosphate-driven reaction producing dUMP, and is finely tuned by thymidylate kinase, which makes its immediate precursor dTDP.

**Figure 2. Synthesis and salvage of pyrimidine nucleotides**

Synthesis of UMP begins with orotate phosphoribosyltransferase followed by decarboxylation (brown arrows). The anabolic pathway ends up with UTP and CTP (green arrows). Salvage of CTP stems from RNA

metabolism (red arrows) and lipid metabolism (purple arrows). Degradation and scavenging of cytosine-based nucleotides goes through uracil-based scavenging and return to the CTP biosynthetic pathway, with cytosine deamination of intermediates as critical steps. Uracil phosphoribosyltransferase matches the role of the orotate counterpart in the ultimate salvage of the base (blue arrow). No counterpart has been yet identified, to our knowledge (see text), for scavenging cytosine (crossed out light blue arrow). Distribution of relevant enzymes in *H. sapiens* and model bacteria is illustrated in **Table 1**.

**Figure 3: Dynamic of base composition at the coding regions of SARS-CoV-2.**

The base composition of the coding regions concatenated by 26 ORFs of SARS-CoV-2 is displayed. Each dot represents one sequence. The calculation was based on 2,574 unique SARS-CoV-2 strains isolated from December 24$^{th}$, 2010 to April 17$^{th}$, 2020.

**Figure 4. Alignment-derived distances of the 89 reference coronavirus genomes represented by cladograms**

A genome-based tree is generated based on the full genome sequence alignment of the group (panel A) and a gap-based tree is created, based on insertions and deletions (indels) only (see Methods). The seven known human coronavirus strains are highlighted by a red colour for the corresponding branches. A panel on the right indicates the four coronavirus groups; in panel B, the two incongruent sub-groups are shown by the same colour code with reduced opacity (alpha and beta sub-groups). For details, please see text.

The 20 SARS-CoV-2 ORFs with a length of over 300 nucleotides were submitted to RSCU calculation. The codon usage for 120,426 human coding regions was also displayed to facilitate comparison. Codons are displayed with the first letter denoting the amino acid and the three letters following a dot representing the codon. The four panels separate the codons according to the nucleotide located at the third codon position. Codons that are not used (RSCU=0), under-represented (RSCU<0.6), normally utilized (RSCU ranges between 0.6-1.6), or over-represented (RSCU>1.6) are labeled in gray, blue, ice cold and yellow, respectively.

## SUPPLEMENTARY MATERIAL

**Supplementary Table S1. List of representative coronaviruses for phylogeny reconstruction and codon usage analysis with ORF1ab a**

**Supplementary Table S2. Acknowledgement Table for the 2,574 SARS-CoV-2 isolates retrieved from GISAID (www.gisaid.org)**

**Supplementary Figure 1: Phylogeny and nucleotide composition of representative coronaviruses.** The Maximum Likelihood phylogeny (left panel) was reconstructed based on the complete genomic sequences. Bootstrap values over 70 are displayed on nodes. Coronaviruses that infects human are highlighted by a red dot at tip. Genus of each strain is indicated by the prefix of the taxa name. The nucleotide composition (right panel) was calculated based on the full genome sequences.

**Supplementary Figure 2: Correlation between base content of SARS-CoV-2 coding regions.** The calculation was based on 2,574 unique SARS-CoV-2 coding sequences concatenated by 26 ORFs.

**Supplementary Figure 3: Details of the codon usage bias of SARS-CoV-2 ORFS viewed from the human tRNA complement.** The X-axis displays codons sorted by amino acid and then by tRNA usage. The Y-axis indicates the mean RSCU value determined for each ORF or the human total coding sequences. Codons using the same tRNA are labeled with the same colour.
